# Association between urinary mixture metal levels and olfactory function in coal miners

**DOI:** 10.3389/fpubh.2024.1447290

**Published:** 2024-09-25

**Authors:** Yang Li, Yuxuan Jiao, Rong Hu, Guilin Hu, Ge Shi, Kaidong Wang, Ai Qi, Yujing Li, Yonghang Li, Zhuoheng Shen, Jiafei Yang, Zhiyun Ha, Yaowen Yang, Jiangping Li, Min Huang

**Affiliations:** ^1^School of Public Health, Ningxia Medical University, Yinchuan, China; ^2^Key Laboratory of Environmental Factors and Chronic Disease Control, Yinchuan, China; ^3^Department of Epidemiology and Health Statistics, School of Public Health, Ningxia Medical University, Yinchuan, China; ^4^The Fifth People’s Hospital of Ning Xia, Shizuishan, China

**Keywords:** Metal mixture, olfaction disorders, coal miners, occupational exposure, UPSIT, Bayesian kernel machine regression, quantile g-computation

## Abstract

**Background:**

Exposure to occupational metallic mixtures has a potential impact on olfactory function. However, research evidence is limited on the potential impact of exposure to metallic mixtures and olfactory dysfunction. Furthermore, the coal dust generated contains multiple various metals during coal mining, and no study yet has focus on the olfactory dysfunction of coal miners.

**Objectives:**

In this study, we evaluate the association between urinary metallic mixtures and olfactory function in coal miners, while also exploring the potential applicability of plasma olfactory marker protein (OMP) as a biomarker for assessing olfaction.

**Methods:**

From July to October 2023, coal workers from seven different coal mining enterprises were recruited for the survey when they come for the employee health checkup. Ultimately, 376 participants were met the inclusion criteria and, respectively, determined with the concentrations of urine (16 metals) and plasma (OMP). Meanwhile, applying UPSIT to access their olfactory function. Binary logistic regression and restricted cubic spline (RCS) model were used to estimate the association of individual metals with olfactory function. Bayesian kernel machine regression (BKMR) and Quantile g-computation (QG-C) regression were employed to assess the overall association between metal mixtures and olfactory function and identify the major contributing elements.

**Results:**

In a single-metal model, two metals in urine were found to be significantly associated with olfactory function. RCS analysis further revealed that the association between Iron (Fe) and olfactory function was linear, while Lead (Pb) exhibited a non-linear. The BKMR model demonstrated a significant positive association between metal mixture concentration and olfactory function. Combined QG-C regression analysis suggested that metals Cr, Fe, Se, Sb, and Pb could impact the performance of the olfactory test (UPSIT), with Pb being identified as the most influential contributor. The correlation between plasma OMP protein levels and urinary metal concentrations was weak.

**Conclusion:**

Multiple metals are associated with olfactory function in the coal miners. A significant positive association was observed between metal mixture concentrations and olfactory function, with Pb being the most important contributor. In this study, plasma OMP has not been demonstrated to serve as a biomarker for olfactory function.

## 1 Introduction

Olfactory function has been associated with impaired appetite and nutrition, decreased quality of life, cognitive impairment, and heightened risk of mortality (1–3). Moreover, olfactory dysfunction (OD) has also been recognized as an early indicator of neurodegenerative diseases such as Alzheimer’s disease (AD) and Parkinson’s disease (4). OD has been regarded as an age-related phenomenon for a long time, however, recent studies has indicated that pollution in the environment are potentially associated with OD (5–7).

Metals are widespread in the environment, and as typically coexist. With the process of industrialization, a multitude of metals are released into the environment and subsequently enter the human body through various pathways among which the first is respiratory (8). The workplace is most frequently associated with exposure to metals. As one of the most crucial energy resources globally, coal significantly contributes to the world economy, but the generation of coal dust throughout the entire mining process poses a significant concern in the field of coal mining (9). Noticeably, the particle size of coal dust typically spans from nanometers to micrometers, and it encompassing various potentially toxic metallic elements, means that coal dust and the metals it carries can get into the olfactory nerve and exert neurotoxicity (10–12).

Coal industry is notorious for a high prevalence of occupational diseases (13). While existing studies have emphasized the health impacts of coal mining on workers, predominantly focusing on elevated rates of pneumoconiosis and other respiratory diseases. However, after considering the anatomical aspects and conducting a comprehensive literature review, there is a scarcity of research specifically addressing the olfactory health of coal miners. For coal workers, a good olfactory function is not only in the fact that smell plays a crucial role in work efficiency and emotional state but also in effectively identifying potentially hazardous substances or odors and safeguarding workplace safety as fast as soon (14). Therefore, it is important to pay attention to the olfactory health of coal miners.

We conducted a literature review and found that only a few studies focused on the relationship between environmental or occupational metals and olfaction. A survey was conducted on workers employed in a cadmium-nickel battery factory, the olfactory dysfunction was diagnosed in approximately 45% of these workers (15). Other metals such as Mn, Zn and olfactory decline has also been revealed (16–18). Furthermore, toxicological studies have demonstrated that metals can induce olfactory dysfunction (19, 20). Although these studies revealed that metals are associated with OD, however, the available evidence is insufficient to establish the impact of metals on olfaction, and these studies only focus on specific metal, no study has yet explored the association between mixed metals and olfactory function.

Hence, the aim of this study is to investigate the olfactory function and urinary metal concentration in coal workers, subsequently elucidating the correlation between mixed metal exposure and olfactory function. To be specific, we aim to assess the potential of plasma olfactory marker protein (OMP) as a biomarker for olfaction. To capture the complexity of mixed exposures. We employed Bayesian kernel machine regression (BKMR) and quantile g-computation (QG-C) mixture models.

## 2 Materials and methods

### 2.1 Study population

The Ningxia Hui Autonomous Region ranks as the eighth largest producer of raw coal in China, boasting abundant coal resources. From July to October 2023, a total of 1900 coal workers were recruited for the survey when they come for the employee health checkup at the Fifth People’s Hospital of the Ningxia Hui Autonomous Region, these workers come from seven different coal mining enterprises in Ningxia, and within the 4 months, the average Air Quality Index is 76.5, the grade is good. Ultimately, 376 individuals met the inclusion criteria for this study. The included individuals were all front-line workers in coal mining enterprises (such as coal miners, underground workers, and coal washing workers), all of whom had direct exposure to coal dust in their work. The inclusion and exclusion criteria were as follows: (1) work experience more than 2 years; (2) no retirement and less than 60 years of age; (3) no history of mental illness, dementia, hearing, or language disorders; (4) no history of severe cardiovascular or respiratory diseases. Additionally, blood and urine samples were collected and stored at minus eighty degrees Celsius for further testing and analysis. All peripheral blood samples were collected by qualified professionals at the examining hospital using sterile disposable materials through venipuncture. A 5 mL peripheral venous blood sample was collected from fasting workers overnight and the serum was separated. Over 30 mL of morning urine samples were collected in sterile centrifuge tubes for metal exposure testing. All samples were obtained within 1 day and stored at −80°C until laboratory analysis.

### 2.2 Urine metals assessment

Dilute the sample 6 times with 1% nitric acid (HNO3), filter, and inject directly for further analysis. The concentrations of urinary metals were detected by the iCAP Qc Inductively Coupled Plasma Mass Spectrometer (ICP-MS, Thermo Fisher Scientific, United States). In this study, 16 metals included aluminum (Al), vanadium (V), lithium (Li), chromium (Cr), manganese (Mn), iron (Fe), cobalt (Co), nickel (Ni), copper (Cu), zinc (Zn), arsenic (As), selenium (Se), molybdenum (Mo), cadmium (Cd), antimony (Sb), lead (Pb)were analyzed.

### 2.3 Olfactory functional assessment

The University of Pennsylvania Smell Identification Test (UPSIT) is utilized for the evaluation of individuals’ olfactory function (21–23). This assessment comprises a set of forty distinct odors, with a maximum attainable score of 40 points. Implemented in a four-option, forced-choice format, the UPSIT necessitates participants to correctly identify each odorant from microencapsulated “scratch and sniff” samples. Grading criteria are established based on age, gender, and scores, classifying individuals into categories such as “normal olfactory function,” “mild olfactory impairment,” “severe olfactory impairment,” and “olfactory loss.”

### 2.4 Blood olfactory marker protein detection

When workers undergo occupational health examinations, fasting cubital venous blood samples are collected. The blood samples are allowed to clot naturally at room temperature for 60 min, then centrifuged at approximately 3,000 xg for about 20 min to collect the serum samples. Individuals with incomplete blood sample information were excluded. Samples were collected, and the plasma was stored at −80°C until further analysis.

The olfactory marker protein (OMP) was thought to be expressed in the olfactory receptor neurons (ORN) in the nasal cavity, and later studies found that it is expressed in mature chemosensory neurons in the olfactory epithelium, serving as a marker for mature olfactory neurons (24–26). Therefore, we used an ELISA kit (EIAABSCIENCEINC, WUHAN, Catalog No: E15888h) to detect the levels of OMP in plasma and to examine its ability to reflect the level of olfactory function.

### 2.5 Statistical analysis

The general demographic data is described using mean ± standard deviation (M ± SD), and differences are compared using t-tests and chi-square tests. The Wilcoxon rank-sum test is used to compare differences in metal and olfactory marker protein concentrations in different populations. Due to the non-normal distribution of metal concentrations, a logarithmic transformation was applied.

Logistic regression and restricted cubic spline (RCS) models are used to estimate the relationship between individual metal elements and olfactory function. Bayesian kernel machine regression (BKMR) and quantile g regression (QG-C) are used to assess the overall association between metal mixtures and olfaction, and to determine the main contributing factors. BKMR offers a flexible approach for modeling the combined effects of mixtures, allowing for potential interactions and non-linear effects. QG-C model can be utilized to examine the positive or negative weights assigned to individual metals, as well as the combined impact of mixed metals. In this study, multivariable linear regression, BKMR, and QG-C models were used to examine the associations between metal mixtures and olfactory function (27, 28). All computations were performed using R 4.3.1. BKMR and QG-C models were fit in R using the ‘bkmr’ and ‘qgcomp’ packages, respectively.

### 2.6 Covariate

**Figure 1:**
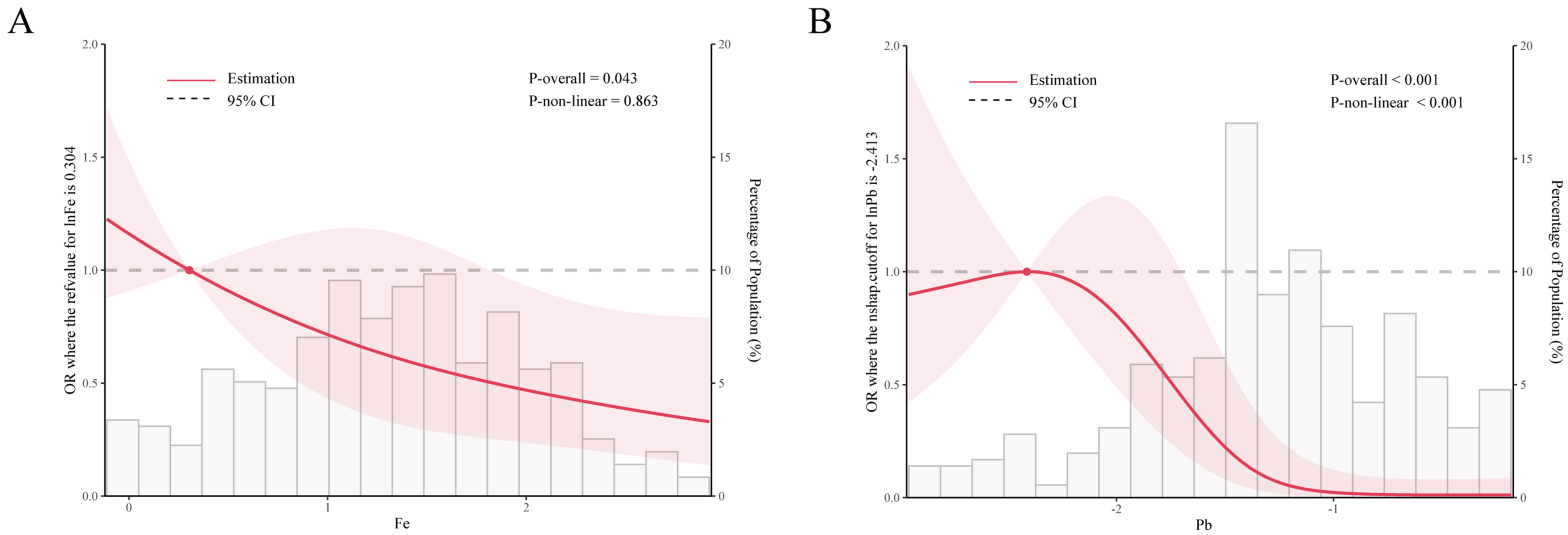
The restricted cubic sample bars for the association of nine urinary metal (Fe **(A)** and Pb **(B)**) concentrations with olfactory function.

We incorporated clinically meaningful covariates in our study, including age, gender, body mass index (BMI), smoking, alcohol consumption, exercise frequency, years of employment, daily working hours, work protection, sleep duration, and sleep quality. According to participants’ self-reports, smoking was categorized as smoking and non-smoking, while alcohol consumption was divided into never, occasional, frequent, and daily. Sleep quality was rated on a scale of 1 to 10, with participants self-assessing their own sleep quality. Exercise frequency was categorized as almost daily, at least once a week, occasional, and never. Work protection referred to whether respiratory protection equipment, such as masks, was worn when performing coal mining-related tasks (Figure 1).

## 3 Results

### 3.1 Coal miners performed worse on UPSIT

The inter-precision (relative standard deviation, RSD) was <5%, and the spike recovery of all elements fell within 83.7 –121.6%. Finally,14 metals with a detection rate > 80% (Al, V, Cr, Fe, Co, Cu, Zn, As, Se, Mo, Cd, Sb, Ba, and Pb) were included in our analysis. But the metallic elements Mn and Ni were excluded because their detection rate was <80% (Supplementary Table S1). Basic characteristics of the workers in this study shown in Table 1. Due to the non-normal distribution of olfactory marker proteins in the blood and metal levels in the urine, their means and quartiles were reported. The average age of the subjects was 48 years all of them worked averagely in coal mine-related occupations for 20 years and the working time was an average of over 9 h per day. Although the average age of the subjects was not high, in the UPSIT test, only 42% showed a degree of “Normal” for smelling, while 14.1% showed olfactory dysfunction, reaching nearly half of the subjects demonstrating varying degrees of olfactory impairment, approaching the incidence rate of olfactory dysfunction in the older adult as mentioned in previous studies.

**Table 1:** Basic characteristics of the workers in this study (*n* = 376).

Variables	
age [mean (SD)]	47.98 (6.28)
**Sex**	
Male (%) Female (%)	355 (94.4%) 22 (5.6%)
Worktime [mean (SD)]	9.35 (2.19)
Work years [mean (SD)]	21.98 (10.44)
Hyper pressure [mean (SD)]	124.53 (19.02)
Lower pressure [mean (SD)] Sleep time [mean (SD)]	91.38 (72.90) 6.80 (1.25)
**Alcohol (%)**	
Almost every day 2–3 times a week A few times a month Never	74 (19.68) 5 (0.01) 168 (44.68) 129 (34.30)
**Smoke (%)**	
Yes	239 (63.6)
No	137 (36.4)
**Olfactory test grade (%)**	
Normal	158 (42.0)
Mild olfactory impairment	82 (21.8)
Server olfactory impairment	83 (22.1)
Olfactory loss	53 (14.1)
UPSIT score [mean (SD)]	29.91 (6.06)
OMP [median (IQR)]	0.98 [0.33, 1.34]
Al [median (IQR)]	3.02 [1.42, 5.84]
V [median (IQR)]	0.03 [0.01, 0.04]
Cr [median (IQR)]	0.33 [0.24, 0.47]
Fe [median (IQR)]	3.93 [2.30, 6.43]
Co [median (IQR)]	0.03 [0.01, 0.06]
Cu (median [IQR])	2.12 [1.48, 3.11]
Zn [median (IQR)]	122.57 [76.53, 179.44]
As [median (IQR)]	7.63 [5.11, 12.04]
Se [median (IQR)]	8.80 [6.07, 12.29]
Mo [median (IQR)]	16.43 [11.06, 24.99]
Cd [median (IQR)]	0.14 [0.05, 0.33]
Sb [median (IQR)]	0.30 [0.26, 0.36]
Ba [median (IQR)]	0.70 [0.51, 1.11]
Pb [median (IQR)]	0.28 [0.19, 0.42]

### 3.2 Fe and Pb in miners‘urine are highly correlated with olfactory dysfunction

Using multiple linear regression to assess the relationship between individual metals and olfactory impairment (Table 2), after fully adjusting for covariates, the results showed a significant correlation between Fe and Pb and decreased olfactory function. Subsequently, RCS model for further analysis the two metals of Fe (*p* = 0.015) and Pb showed statistically significant trends in the single-metal model. After fully adjusting for covariates, it was found that Fe was linearly correlated with olfactory function (*P* non-linear > 0.05), and as the concentration of Pb in the urine increased, the incidence of olfactory dysfunction also gradually increased, with a turning point on the *Y*-axis. As the concentration of urinary Fe increased, the incidence of olfactory dysfunction also gradually increased. The relationship between urinary Pb and the incidence of olfactory dysfunction formed an “L-shaped” curve, with the incidence of olfactory dysfunction gradually increasing as the concentration of urinary Pb increased, eventually stabilizing.

**Table 2:** Correlation of individual metals and olfactory function.

	Crude model	Adjusted model
Al	1.60 (1.30–1.97, *p* < 0.001)	1.21 (0.95–1.54, *p* = 0.123)
V	1.20 (1.00–1.44, *p* = 0.056)	1.00 (0.79–1.26, *p* = 0.970)
Cr	1.63 (1.16–2.29, *p* = 0.005)	1.07 (0.72–1.59, *p* = 0.724)
Fe	2.03 (1.52–2.73, *p* < 0.001)	1.51 (1.08–2.10, *p* = 0.015)
Co	1.08 (0.93–1.25, *p* = 0.326)	0.99 (0.83–1.18, *p* = 0.930)
Cu	2.17 (1.48–3.20, *p* < 0.001)	1.18 (0.75–1.84, *p* = 0.473)
Zn	1.17 (0.90–1.52, *p* = 0.231)	1.03 (0.76–1.38, *p* = 0.865)
As	1.67 (1.25–2.24, *p* < 0.001)	1.27 (0.93–1.73, *p* = 0.126)
Se	1.92 (1.36–2.73, *p* < 0.001)	1.32 (0.94–1.86, *p* = 0.104)
Mo	1.43 (1.05–1.94, *p* = 0.023)	1.08 (0.75–1.55, *p* = 0.697)
Cd	1.43 (1.19–1.71, *p* < 0.001)	1.17 (0.96–1.43, *p* = 0.130)
Sb	1.46 (1.13–1.90, *p* = 0.004)	1.28 (0.98–1.68, *p* = 0.074)
Ba	1.64 (1.15–2.34, *p* = 0.006)	1.02 (0.65–1.60, *p* = 0.923)
Pb	4.81 (3.08–7.51, *p* < 0.001)	2.54 (1.14–5.66, *p* = 0.023)

^Crude Models have not been adjusted in any way. Adjusted model adjusted for sex, age, education, work time, BMI, sleep time, smoke, alcohol, exercise, blood pressure.^

### 3.3 Metal mixture and olfactory function correlation analysis

**Figure 2:**
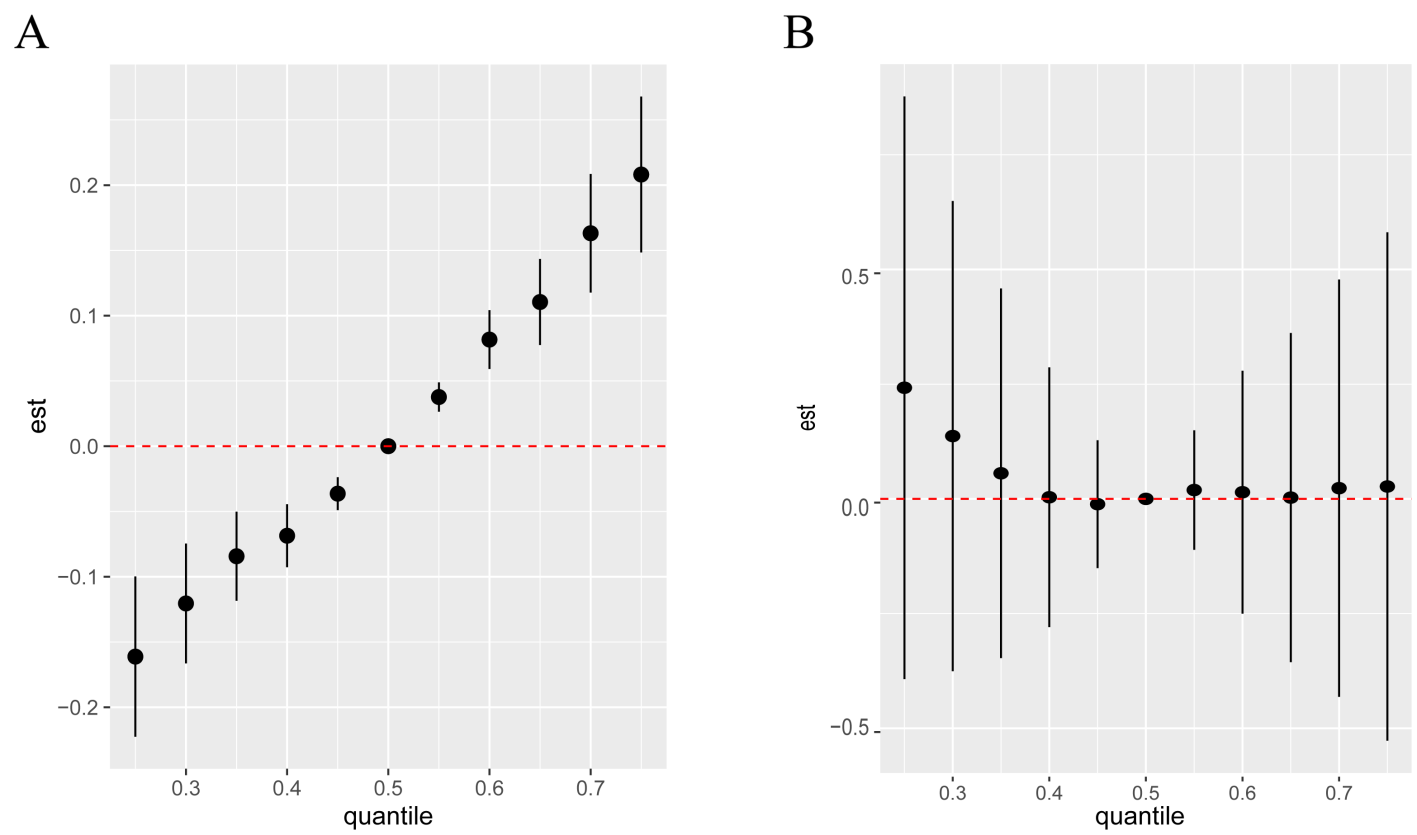
Associations between the overall metal mixture and UPSIT **(A)** and OMP **(B)** by Bayesian kernel machine regression analyses.

Using BKMR to assess the overall association between metal mixtures and olfactory function (Figure 2). We found that after fully adjusting for covariates, the overall trend decreased with increasing concentration of the metal mixture, showing a significant positive correlation (Figure 2A). However, there was only a weak trend in the association between mixed metal exposure and blood OMP, which was not statistically significant (Figure 2B). The “univariate-response function plot” shows the relationship between the concentration of each metal and olfactory function when the concentrations of other metals are fixed at the median level. We found that as the concentrations of Al, Cr, Se, and Sb increased, their overall association with decreased olfactory function tended to increase (Figure 2A). Pb showed an inverted U-shaped correlation with olfactory function, with a positive trend, and the posterior inclusion probability of urinary Pb was the highest (PIP value was 1 in Table 3), indicating its significant role in the correlation between urinary metals and olfactory function. Figure 2B shows that there is a non-linear correlation between urine levels of Al, Fe, Cd, and blood levels of OMP. Al shows a negative correlation trend with blood OMP levels, as urine Fe levels increase, blood OMP levels gradually decrease and later stabilize, while the correlation trend between urine Cd and blood OMP is negative. Urine levels of Cr, Pb, and Ba gradually increase, and blood OMP levels also increase; Se and Sb are negatively correlated with blood OMP. Fe and Cd have the highest posterior inclusion probability (PIP value of 1, Table 3).

**Table 3:** PIPs obtained using the BKMR model for each metal with olfactory function.

Variable	PIP1	PIP2
Al	0.02088	0.8336
V	0.05032	0.8428
Cr	0.01056	0.812
Fe	0.00768	1
Co	0.01504	0.5028
Cu	0.01184	0.6368
As	0.00688	0.7592
Se	0.10168	0.6504
Mo	0	0.624
Cd	0.0024	1
Sb	0.0544	0.5472
Ba	0.01048	0.63
Pb	1	0.6436
Zn	0.03552	0.6848

To fully explore the potential interaction with the metal mixture, Bayesian kernel machine regression model was used. In this model, all other elements were fixed at different quartiles (25th, 50th, 75th), the exposure effect of individual metals on olfactory function and its potential biomarkers was analyzed, and it was found that the level of Pb was highly correlated with decreased olfactory function.

**Figure 3:**
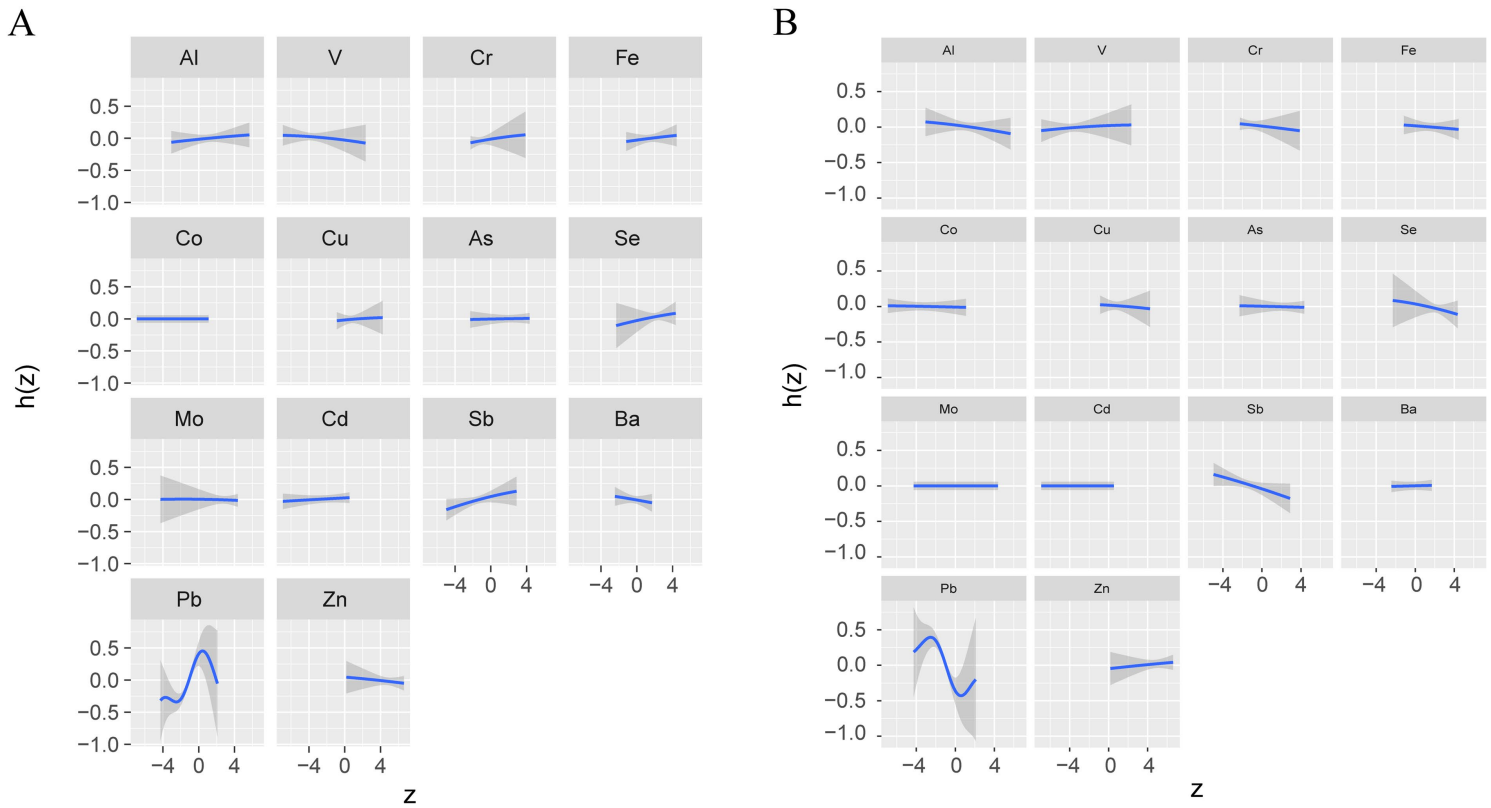
Univariate exposure-response functions of each metal (95% confidence intervals) and UPSIT **(A)** and OMP **(B)** Bayesian kernel machine regression analyses.

To visualize potential pairwise interactions, we plotted bivariate exposure-response functions of one element with olfactory function at different quartiles (25th, 50th, 75th) of a second element when all other element exposures are set to their median (Supplementary Figure S1), due to only the overall effect of mixed metals and olfactory function is significant (Figure 2A), we focus only on the UPSIT results, and we found Pb was significantly associated with UPSIT. To visualize potential pairwise interactions, we plotted bivariate exposure-response functions of one element with UPSIT at different quartiles (25th, 50th, 75th) of a second element when all other element exposures are set to their median (Supplementary Figure S2), we found that when the concentrations of Al, Cd, and Fe are fixed at the 25th, 50th, and 75th percentiles, they all significantly affects the relationships between other metals and olfactory function, specifically manifested in influencing the slope and shape of the correlation curve (Figure 3).

### 3.4 QG-C analysis of the correlation between various metal elements and olfactory dysfunction

**Figure 4:**
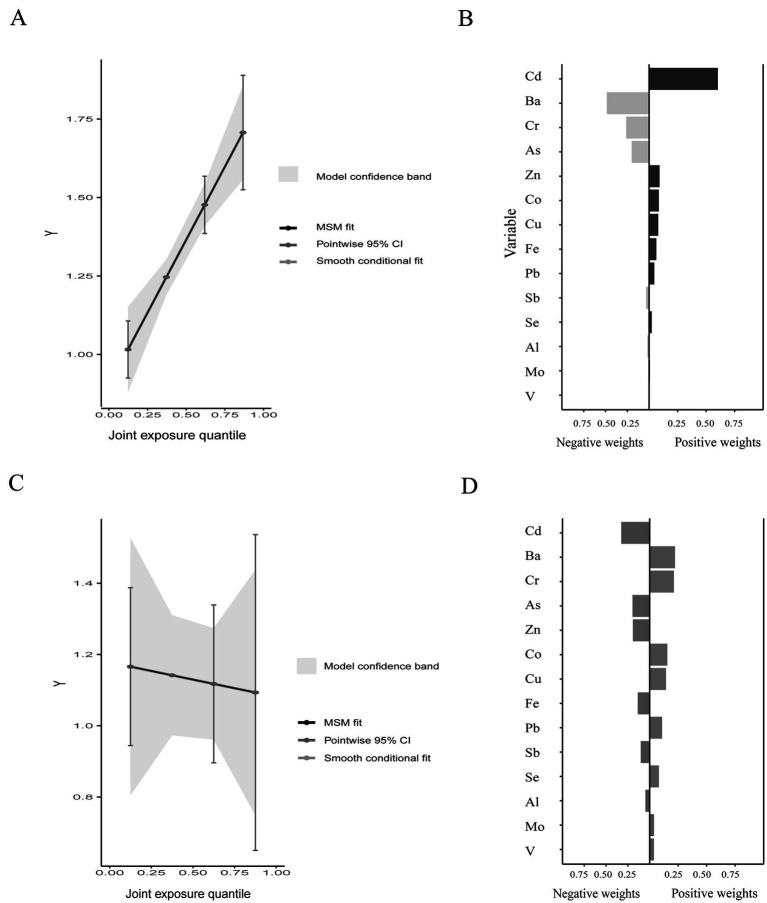
The joint effects of metal mixtures on olfactory function and plasma OMP Quantile g-computation analyses **(A,C)** and weight of each metal in the association by Quantile g-computation analyses **(B,D)**.

In the QG-C model, the occurrence rate of olfactory dysfunction is positively correlated with the metal mixture (Figure 4A) and has a weak negative correlation with blood OMP protein levels, it can be considered that as the concentration of the metal mixture increases, the occurrence rate of olfactory dysfunction increases, and the plasma OMP levels show a decreasing trend (Figure 4C). Pb has the largest positive weight in the occurrence of olfactory dysfunction, while Ba, Cu, and Zn are assigned relatively large negative weights (Figure 4B). Ba, Cr, Co, and Cu have relatively large positive weights in the negative correlation between OMP and urinary metals, while Cd, As, and Zn have relatively large negative weights (Figure 4D).

### 3.5 OMP of the blood does not serve as a potential marker for olfactory dysfunction

To investigate the relationship between plasma OMP levels and olfactory function, we used logistic regression model to examine the association between plasma OMP levels and UPSIT test results. Here, the UPSIT test results were divided into two categories, namely whether the olfactory function is normal. Unfortunately, we did not obtain the desired results (*p* > 0.05).

## 4 Discussion

Our study establishes a connection between occupational metal exposure and olfactory health. The association between a mixture of urinary metals and olfactory function was analyzed in this study using multivariate linear regression, Bayesian kernel machine regression (BKMR), and the Quantile g-computation (QG-C) model. After adjusting for covariates in the generalized linear regression model, significant effects of Fe and Pb on olfactory function were observed. BKMR and QG-C models were employed to investigate the relationship between urinary multi-metals and olfactory decline, revealing a positive correlation between levels of mixed metals in urine and olfactory decline. Higher individual urinary metal levels were associated with an increased likelihood of experiencing olfactory decline. Furthermore, as concentrations of Al, Cr, Se, and Sb increased, their overall association with olfactory decline tended to strengthen. Notably, Pb emerged as the most influential contributor. We also assessed plasma OMP levels in human subjects and observed a weak inverse correlation between plasma OMP and urinary mixed metal concentrations, while no significant association was found between plasma OMP and olfactory function.

Our study reveals the detrimental effect of iron (Fe) on olfactory function. Iron, as an important trace element involved in numerous fundamental biological processes in the brain, is also a prominent metal associated with neurodegeneration and neurodegenerative diseases (29). The accumulation of iron in the brain may lead to a range of neurodegenerative symptoms. There is no iron in the brain at birth, and it rapidly increases between youth and middle age, remaining relatively stable thereafter (30). With age, the accumulation of iron primarily occurs in the basal ganglia and other brain regions associated with motor function, cognitive function, and visual function (31, 32). Although the specific mechanism is not yet clear, it has been confirmed that lead (Pb) can exacerbate symptoms of allergic rhinitis in both humans and mice (33). The concentration of Zn in urine was found to be significantly higher compared to other metals. In Binggan Wei’s study, the residents in the control area exhibited a significantly lower concentration of Zn in their urine compared to those in the experimental area who were exposed to a high As concentration water source (34). We selected the studies as controls for our study, wherein we observed that the urinary concentration of zinc was also higher compared to the control group. Furthermore, we identified an elevated urinary molybdenum concentration in coal miners. Importantly, it should be noted that high concentrations of zinc and molybdenum in the body may have detrimental effects on the nervous system, leading to symptoms such as headaches, fatigue, and impaired cognitive function. Additionally, there is evidence suggesting a possible association between high levels of zinc in the body and Alzheimer’s disease onset (35, 36). Therefore, coal miners should pay more attention to the concentration of zinc and molybdenum in their bodies, as otherwise it may increase the likelihood of experiencing difficulties in their post-retirement life.

There is one point unfortunately, our study did not establish a significant association between plasma OMP levels and olfactory function due to the inherent complexities of this relationship. Olfactory chemoreception depends on a large multigene family of olfactory specific G-protein coupled receptors (GPCRs) that were initially identified in the rat and are now referred to as the OR family (37). OMP expression can as an indicator of potential OR-associated events in non-olfactory tissues, but it has only been confirmed that OMP immunohistochemical analysis is a useful tool for identifying expression of ORs, suggesting OMP expression is an indicator of potential OR-mediated chemoreception in non-olfactory systems (24–26). Therefore, the selection of plasma OMP as a target presents an initial challenge.

This study possesses several strengths. Firstly, the selection of metals as exposure variables based on the high metal content in coal dust enables an investigation into the impact of primary exposure experienced by coal miners on health outcomes. Secondly, unlike previous studies that primarily focus on pulmonary diseases among coal miners, this study focuses on olfactory function, which has been neglected in past. Thirdly, to our knowledge, this is the first study to explore the effects of mixed metal exposure specifically on the olfactory system. Moreover, this study evaluates the potential of olfactory marker protein expressed in blood as a novel biomarker for assessing olfactory function.

There are several limitations in this study. First, it should be noted that this is a cross-sectional study with limited ability to establish causality. Secondly, although traditional methods such as urine and blood analysis are used to assess trace-element levels in the human body, they may not accurately reflect accumulated levels and metabolism due to variations in pharmacokinetic properties among different metals. Additionally, measuring metal concentration in a single spot urine sample may lead to misclassification of exposure. However, if there have been no significant changes in living environment and lifestyle among our study subjects over the past 10 years, it can be assumed that their metal exposure level would remain stable during this period. It’s also important to note that urinary exposure levels in occupational populations may differ from actual environmental exposures. Thirdly, our study had a relatively small sample size which limits precision in association estimates and may affect generalizability of results. Finally, we did not select an uncontaminated population as a control group in our study, and we plan to address this in future research.

## 5 Conclusion

In summary, our study shows a high detection rate of olfactory abnormalities in coal miners, and Fe and Pb were significantly associated with olfactory dysfunction in coal miners. Furthermore, mixed metals in urine were positively correlated with olfactory decline. Some metals, such as Cr, Fe, Se, Sb, and Pb can affect the performance of olfactory test (UPSIT), and Pb is the most important contributor.

Protecting the occupational health of coal miners must begin with addressing exposure to occupational pollution. During this research, I discovered that some workers still lack the habit and awareness of wearing masks while on the job. Therefore, I believe the first priority should be for enterprises to enforce the correct usage of personal protective equipment (PPE) among workers, complemented by appropriate reward and disciplinary measures. Furthermore, companies should ensure that workers maintain personal hygiene after their shifts, which may include the use of nasal rinses and other hygiene equipment. Additionally, it is crucial to establish a robust occupational health monitoring system that includes regular health examinations with a comprehensive range of assessment items. The frequency of health checks for high-risk positions should be increased accordingly to ensure worker safety and well-being.

## Data Availability

The raw data supporting the conclusions of this article will be made available by the authors, without undue reservation.

## Author’s note

We would like to declare on our behalf that the work described was original and has not been published elsewhere before, in whole or part. All the authors listed have no conflicts of interest and have approved the manuscript that is enclosed.
