# Supplementary material for: Association between urinary mixture metal levels and olfactory function in coal miners
Source: Front Public Health. 2024 Sep 25;12:1447290. doi: 10.3389/fpubh.2024.1447290 (PMC11461330; doi:10.3389/fpubh.2024.1447290)
Supplement: Supplementary file 1 [file Data_Sheet_1.docx]

| Metals | Unit | Detection Rate（%） |
| --- | --- | --- |
| Al | ug/L | 97.16 |
| V | ug/L | 93.10 |
| Cr | ug/L | 100.00 |
| Mn | ug/L | 64.91 |
| Fe | ug/L | 99.39 |
| Co | ug/L | 81.74 |
| Ni | ug/L | 38.54 |
| Cu | ug/L | 100.00 |
| Zn | ug/L | 100.00 |
| As | ug/L | 99.59 |
| Se | ug/L | 99.80 |
| Mo | ug/L | 100.00 |
| Cd | ug/L | 95.13 |
| Sb | ug/L | 88.84 |
| Ba | ug/L | 100.00 |
| Pb | ug/L | 97.36 |

**Table S1**

The metals detection rate


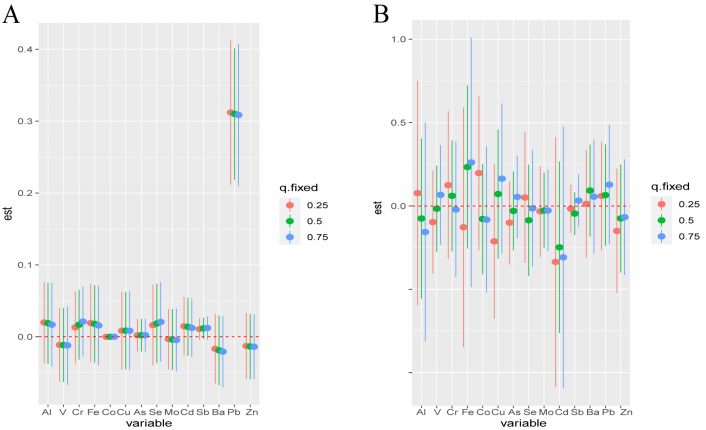


**Fig S1.** The single-exposure effect of individual metals on the UPSIT (A) and OMP (B) (estimates and 95% confidence intervals)when all the other metals are fixed at their 25th, 50th, or 75th percentile by Bayesian kernel machine regression analyses.


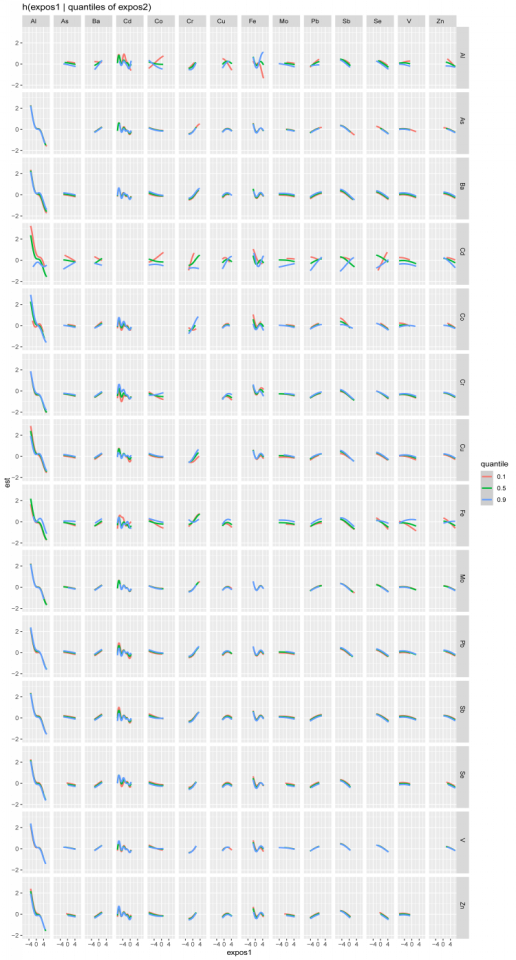


**Fig S2.** Bivariate exposure-response functions of one metal with olfactory function(UPSIT) at different quartiles (25th, 50th, 75th) of a second metal when all other metals are set to their median by Bayesian kernel machine regression analyses.
